# Renal hyperfiltration, fatty liver index, and the hazards of all-cause and cardiovascular mortality in Finnish men

**DOI:** 10.4178/epih.e2021001

**Published:** 2020-12-24

**Authors:** Mounir Ould Setti, Ari Voutilainen, Tomi-Pekka Tuomainen

**Affiliations:** Department of Public Health, University of Eastern Finland, Kuopio, Finland

**Keywords:** Mortality, Cardiovascular diseases, Heart disease risk factors, Glomerular filtration rate, Fatty liver, Non-alcoholic fatty liver disease

## Abstract

**OBJECTIVES:**

Renal hyperfiltration (RHF) and fatty liver are separately associated with adverse health outcomes. In this study, we investigated the mortality hazard of coexisting RHF and fatty liver.

**METHODS:**

Middle-aged men from the Kuopio Ischaemic Disease Risk Factor Study (n=1,552) were followed up for a median of 29 years. Associations among RHF, fatty liver index (FLI) score, age, body mass index, smoking status, alcohol consumption, and hypertension status were assessed using logistic regression. Cox proportional hazards models were used to determine the hazard ratios (HRs) for all-cause and cardiovascular disease (CVD) mortality with respect to RHF and fatty liver.

**RESULTS:**

Of the men, 5% had RHF (n=73), whereas a majority had fatty liver (n=848). RHF was associated specifically with smoking, and fatty liver was associated specifically with overweight. The all-cause mortality hazard was highest (HR, 1.96; 95% confidence interval [CI], 1.27 to 3.01) among men with RHF and fatty liver (n=33). Among men with RHF but normal FLI (n=40), the HR of all-cause mortality was 1.67 (95% CI, 1.15 to 2.42). Among men with fatty liver but a normal estimated glomerular filtration rate (n=527), the HR of all-cause mortality was 1.35 (95% CI, 1.09 to 1.66). CVD mortality hazard was associated with RHF, but not fatty liver. We detected no interaction effect between RHF and fatty liver for all-cause (synergy index, 0.74; 95% CI, 0.21 to 2.67) or CVD (synergy index, 0.94; 95% CI, 0.34 to 2.60) mortality.

**CONCLUSIONS:**

RHF and fatty liver are independently associated with all-cause and CVD mortality

## INTRODUCTION

Chronic kidney disease (CKD) and chronic liver disease (CLD) are becoming increasingly prevalent. As populations have aged, the global prevalence of CKD has increased by nearly 30% since 1990 to affect over 9% of the world’s population and cause 4.6% of global deaths in 2017 [1]. Despite being a well-established risk factor for cardiovascular disease (CVD) mortality even in its early stages [2], CKD has remained an underdiagnosed condition, causing delays in treatment and worsened outcomes [3,4]. Typically, CKD refers to impaired kidney function and a low glomerular filtration rate. However, an abnormally elevated glomerular filtration rate, known as renal hyperfiltration (RHF), also appears to be an early sign of CKD and a predictor of mortality, CVDs, and diabetes [5]. Thirteen of the 15 studies reviewed by Kanbay et al. [6] suggested a strong association between RHF and mortality.

Among contributors to CLD, fatty liver disease (FLD) is the most rapidly growing in prevalence and attributed mortality [7]. The incidence of FLD is correlated with the worldwide spread of obesity and diabetes, which play key roles in the pathogenesis of the disease [8,9]. The hepatic accumulation of lipids causes liver abnormalities, clinically classified as alcoholic and non-alcoholic FLD [10]. The global prevalence of non-alcoholic FLD is 25% and growing [11,12]. Despite the role of FLD in steatogenesis and hepatic cancer, the majority of deaths attributed to non-alcoholic FLD are due to CVD [11]. Like RHF, FLD is common and underdiagnosed [13,14].

RHF and FLD have been presented as independent risk factors for CVD [2,15]. However, as they share common pathogenetic pathways (notably cardiometabolic syndrome [16]), they can coexist and may interact biologically [17,18]. Whether an interaction exists between RHF and FLD with respect to mortality risk has not been studied. In this study, we investigated the combined effect of RHF and fatty liver on the hazards of all-cause and CVD mortality.

## MATERIALS AND METHODS

### Data source

Middle-aged men participating in the Kuopio Ischaemic Heart Disease Risk Factor Study (KIHD) served as our study population. The KIHD includes 2,682 randomly-sampled Finnish men who lived in the city of Kuopio or its surrounding areas between March 1984 and December 1989 [19]. Since then, the KIHD study has followed the men’s health status via the annual review of electronic health records, including the cause-of-death registry administered by Statistics Finland (License TK-53-1770-16) and the Care Register for Healthcare, administered by the National Institute for Health and Welfare (License THL/93/5.05.00/2013).

For this study, we excluded men with diabetes (n=162) as well as those who reported abstaining from drinking alcohol at baseline (n=366). In the KIHD study, men who reported at baseline that they had not consumed alcohol during the previous 12 months differed from other study participants with respect to typical covariates, such as marital status, work status, education level, residential area, smoking status, and overall health [20]. For statistical reasons, we excluded 2 outliers and 600 men with missing values. After exclusions, 1,552 men were included in this study. The median follow-up time was 29 years, and the maximum follow-up time was 34 years. No participants were lost to follow-up.

### Variable measurement

We calculated estimated glomerular filtration rate (eGFR; units of mL/min/1.73 m^2^) based on serum creatinine concentrations by applying the Chronic Kidney Disease Epidemiology Collaboration (CKD-EPI) equation, which adjusts creatinine values for age, gender, and ethnicity [21]. As the KIHD study involved the use of the Jaffe method to measure creatinine concentrations, we multiplied the original creatinine values by 0.95 before the eGFR calculations [22]. We defined the cut-off value between normal and low eGFR based on age-adjusted Finnish guidelines for the normal range of CKD-EPI eGFR [23]. As a cut-off value for RHF, we used the 95th age-adjusted percentile of eGFR [6].

We used the equation described by Bedogni et al. [24] to calculate the FLI score. The equation takes into account body mass index (BMI), waist circumference, serum triglyceride, and serum gamma-glutamyl transferase concentrations to indicate the presence or absence of fatty liver. We considered FLI values < 30 to be normal and values ≥ 30 to be indicative of fatty liver [25].

Salonen et al. [26] described the KIHD study procedures for collecting, processing, and analyzing blood specimens.

We included the following variables as covariates: age, BMI, smoking status (current smoker, previous smoker, or never smoker), alcohol consumption in g/wk, and whether the participant had a diagnosis of hypertension at baseline. These factors are associated with both RHF [27] and adverse cardiovascular outcomes. A KIHD research nurse measured the men’s height, weight, and blood pressure. The men self-reported their smoking habits, alcohol consumption, illnesses, and medications using structured questionnaires. Each man also underwent a physical examination conducted by a physician. The examination included an interview regarding medical history.

The outcomes of interest were all-cause and CVD mortality. The cause-of-death registry provided individual mortality data. CVD deaths were indicated by International Statistical Classification 10th revision codes I00-I99 as an underlying cause of death [28].

### Statistical analysis

First, we used the Kruskal-Wallis test by ranks and the chi-square test for comparisons of baseline characteristics across 6 groups formed based on eGFR and FLI categories. We used the Mann-Whitney U-test and chi-square test for comparisons between survivors and non-survivors.

Second, we used a logistic regression analysis to study associations across RHF, fatty liver, and covariates. We reported the results of the logistic regression as odds ratios (ORs) with 95% confidence intervals (CIs).

Third, we used Cox proportional hazards regression to analyze the associations of eGFR and FLI with all-cause and CVD mortality. We defined the periods at risk for each study participant in days from baseline until death or December 31, 2018. We reported the Cox regression results as hazard ratios (HRs) with 95% CIs and computed the area under the curve and its related 95% CI using the R package riskRegression version 2020.02.05 (https://CRAN.R-project.org/package=riskRegression) to assess the discriminative accuracy of the Cox models. Finally, we computed the synergy index [29] to evaluate, on an additive scale, the relative excess hazard of mortality attributed to the interaction between RHF and fatty liver in the Cox proportional hazards models [30,31]. We computed the 95% CIs of the synergy indices according to Hosmer and Lemeshow [32]. A synergy index of 1 indicates a lack of interaction.

In the analyses, the normal eGFR and normal FLI served as reference categories.

We used R version 4.0.2 (https://www.R-project.org) for all computations and applied the R package Survival Analysis version 3.1-12 (https://CRAN.R-project.org/package=survival) to build the models, test the proportional hazards assumptions with Schoenfeld residuals, and perform the analyses.

### Ethics statement

Ethical approval was obtained from the University of Kuopio Cause of Death Registry authorization (No. TK-53-1770-16). All KIHD study participants provided informed consent.

## RESULTS

Of the 1,552 study participants, 919 (59.2%) died during 34 years of follow-up, and 406 (44.2%) of those deaths were attributed to CVD. The non-survivors tended to be older (median age, 54.5 vs. 48.9 years; p<0.001), to have a higher BMI (median, 26.7 vs. 25.8 kg/m^2^; p<0.001), and to consume more alcohol (median, 47 vs. 37 g/wk; p=0.001) than the survivors, and they had a higher median FLI score (38.2 vs. 28.7; p<0.001). The group of men who died also included more current smokers (42.8 vs. 22.6%, p<0.001) and a greater proportion of people with hypertension (32.4 vs. 22.6%, p<0.001). The median eGFR did not differ significantly between non-survivors and survivors (85.2 vs. 84.0 mL/min/1.73 m^2^; p=0.603).

Fewer than 5% of the studied men were categorized as having RHF (n=73), whereas nearly 55% of them had fatty liver (n=848). RHF and fatty liver coexisted in only 2.1% of the men (n=33). All of the measured baseline characteristics differed significantly across the groups defined according to the eGFR and FLI categories (Table 1).

In the logistic regression analysis (Table 2), RHF was associated specifically with current smoking (OR, 3.44; 95% CI, 1.75 to 7.42), whereas fatty liver was associated specifically with overweight and obesity. RHF and fatty liver were not associated with each other.

**Figure 1.**
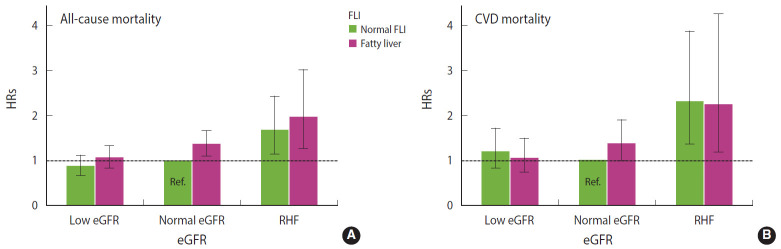
Fully adjusted hazard ratios (HRs) with 95% confidence intervals for (A) all-cause and (B) cardiovascular disease (CVD) mortality with respect to kidney (eGFR) and liver (FLI) functions. eGFR, estimated glomerular filtration rate; RHF, renal hyperfiltration; FLI, fatty liver index; Ref, reference.

In the Cox regression (Figure 1), the HR for all-cause mortality was highest among men with coexisting RHF and fatty liver (HR, 1.96; 95% CI, 1.27 to 3.01). Among men with RHF but normal FLI, the HR for all-cause mortality was 1.67 (95% CI, 1.15 to 2.42). Among men with fatty liver but normal eGFR, the HR for all-cause mortality was 1.35 (95% CI, 1.09 to 1.66). Low eGFR was not associated with an increased hazard of all-cause mortality, and the FLI score was not related to the strength of the association between low eGFR and all-cause mortality (Table 3). The hazard of CVD mortality was highest among men with RHF irrespective of the FLI score (Figure 1 and Table 3).

With synergy indices of 0.74 (95% CI, 0.21 to 2.67) for CVD mortality and 0.94 (95% CI, 0.34 to 2.60) for all-cause mortality, the interaction between RHF and fatty liver was not associated with a change in mortality hazard.

Regarding covariates, being older, consuming more alcohol, being a current smoker, and having hypertension increased the hazards of all-cause and CVD mortality in the Cox regression model (Table 3). Overweight and obesity only increased the hazard of CVD mortality (Table 3).

The accuracy of the Cox regression model for all-cause mortality was 75.1%, and that of the model for CVD mortality was 74.5% (Supplementary Material 1).

## DISCUSSION

In this study, we evaluated the hazards of long-term all-cause and CVD mortality in a cohort of middle-aged Finnish men in relation to different categories of eGFR and FLI. This study showed that the coexistence of RHF and fatty liver was associated with a higher HR for all-cause mortality than either of the conditions alone. However, we did not find an interaction between RHF and fatty liver, and the coexistence of the 2 conditions was not associated with a synergic or an antagonistic effect on mortality. Regarding CVD mortality, a high HR was associated with RHF irrespective of fatty liver status. Moreover, the study suggested that RHF and fatty liver are not associated with each other per se; rather, RHF relates specifically to smoking and fatty liver relates to obesity.

In general, evidence exists of the relationship between CKD and FLD; non-alcoholic FLD may increase the risk of CKD and other diseases typically related to health behaviors [15,33-36]. In the present study, we did not find an association between RHF and fatty liver, strengthening the view that CKD and FLD may be completely independent of each other in their associations with mortality. Previously, Paik et al. [37] demonstrated that CKD and non-alcoholic FLD are independently associated with increased mortality. Similarly to the global figures [38], RHF and fatty liver coexisted among only a low percentage of the KIHD participants.

Like Maeda et al. [39], we found a strong association between tobacco smoking and RHF. According to Park et al. [40], smoking is one of the most important covariates to consider when analyzing the association between RHF and mortality. Our study underlines the recommendation by Park et al. [40]. In addition to smoking, controlling for BMI in RHF studies is of particular importance, as obesity and low muscular mass tend to affect the accuracy of eGFR [6,41].

In our study, low eGFR category was not associated with mortality risk (Table 3). A possible reason for this observation is that the eGFRs of most of our participants in the low-eGFR category were closer to the normal values than to the values that indicate chronic kidney disease (interquartile range, 68-75 mL/min/1.73 m^2^).

As a limitation, we acknowledge that our findings represent only middle-aged men. While this fact hinders the generalizability of our findings, most chronic diseases start appearing in middle age [42], not earlier, and both RHF [43] and FLD [44] are more common in men than women. In addition to age and gender, the homogenous ethnicity and regional nature of our study population represent another limitation to the generalizability of our results.

## Figures and Tables

**Table 1.** Baseline characteristics of the study population by kidney (eGFR) and liver (FLI) function

Characteristics		Total	Normal eGFR-		Low eGFR-		RHF		*p*-value^1^
			Normal FLI	Fatty liver	Normal FLI	Fatty liver	Normal FLI	Fatty liver	
Total (%)		1,552 (100)	474 (30.5)	527 (33.9)	190 (12.2)	288 (18.5)	40 (2.6)	33 (2.1)	
Deaths (column %)		919 (59.2)	254 (53.6)	351 (66.6)	81 (42.6)	177 (61.4)	32 (80.0)	24 (72.7)	<0.001
CVD deaths (column %)		406 (26.1)	98 (20.7)	158 (30.0)	42 (22.1)	80 (27.8)	17 (42.5)	11 (33.3)	0.001
Age (yr)		54.4 [48.4, 54.8]	54.4 [48.3, 55.0]	54.5 [48.8, 60.2]	54.4 [48.3, 54.5]	54.4 [48.7, 54.8]	54.3 [48.3, 54.5]	54.4 [48.2, 54.9]	0.003
BMI (kg/m^2^)		26.4 [24.5, 28.7]	24.5 [23.1, 25.8]	28.1 [26.6, 30.4]	24.5 [23.0, 25.7]	28.6 [26.8, 30.5]	24.5 [23.2, 25.6]	26.6 [25.2, 29.5]	<0.001
Smoking status (column %)									<0.001
	Never smokers	458 (29.5)	147 (31.0)	133 (25.0)	85 (45.0)	83 (29.0)	6 (15.0)	4 (12.0)	
	Previous smokers	558 (35.9)	132 (27.8)	209 (40.0)	65 (34.0)	130 (45.0)	7 (18.0)	15 (45.0)	
	Current smokers	536 (34.5)	195 (41.0)	185 (35.0)	40 (21.0)	75 (26.0)	27 (68.0)	14 (42.0)	
Alcohol consumption (g/wk)		42.6 [12.4, 105.1]	27.9 [9.6, 72.7]	54.9 [22.0, 125.0]	33.4 [10.2, 86.0]	51.0 [17.0, 138.6]	48.0 [11.9, 128.4]	76.0 [41.5, 197.0]	<0.001
Hypertension (%)		441 (28.4)	87 (18.0)	193 (37.0)	39 (21.0)	106 (37.0)	10 (25.0)	6 (18.0)	<0.001
eGFR (mL/min/1.73 m^2^)		85.0 [76.3, 95.4]	72.4 [68.3, 75.8]	71.9 [67.4, 75.3]	89.8 [83.6, 96.8]	88.6 [83.9, 96.9]	107.5 [104.8, 111.1]	108.6 [105.9, 113.6]	<0.001
FLI		33.8 [17.3, 57.4]	16.2 [11.9, 23.3]	53.2 [40.4, 73.0]	15.9 [9.4, 23.0]	54.3 [42.4, 73.7]	14.5 [9.7, 23.8]	52.6 [36.2, 70.4]	<0.001
Follow-up in years		28.9 [20.1, 31.1]	29.8 [22.1, 31.4]	28.3 [21.3, 30.5]	29.7 [21.7, 31.7]	26.7 [18.3, 30.4]	22.6 [14.9, 30.1]	23.4 [12.2, 29.8]	<0.001

Values are presented as number (%) or median [interquartile range]. eGFR, estimated glomerular filtration rate; FLI, fatty liver index; RHF, renal hyperfiltration; CVD, cardiovascular disease; BMI, body mass index.

1 The Kruskal-Wallis rank-sum test and the chi-square test were used for across-groups comparisons.

**Table 2.** RHF and fatty liver with respect to baseline characteristics in the study population (n=1,552)

Characteristics	RHF	Fatty liver
Age in years	1.00 (0.96, 1.04)	1.01 (0.98, 1.03)
Normal weight	1.00 (reference)	1.00 (reference)
Slightly overweight	0.71 (0.39, 1.29)	7.14 (5.18, 9.95)^**^
Overweight	0.46 (0.19, 1.07)^†^	57.45 (36.79, 92.31)^**^
Obese	0.29 (0.09, 0.81)^*^	∞ (all obese)
Alcohol consumption (100 g/wk)	1.05 (0.87, 1.18)	1.46 (1.27, 1.69)^**^
Never smoker	1.00 (reference)	1.00 (reference)
Previous smoker	2.00 (0.95, 4.50)^†^	1.50 (1.05, 2.14)^*^
Current smoker	3.44 (1.75, 7.42)^**^	1.41 (0.98, 2.02)^†^
Hypertension	0.84 (0.45, 1.47)	1.44 (1.05, 1.98)^*^
Normal FLI	1.00 (reference)	-
Fatty liver	1.10 (0.59, 2.03)	-
Normal eGFR	-	1.00 (reference)
Low eGFR	-	1.60 (1.18, 2.19)^**^
RHF	-	1.24 (0.66, 2.30)

Values are presented as adjusted odds ratio (95% confidence interval). eGFR, estimated glomerular filtration rate; FLI, fatty liver index.

† p<0.1,

* p<0.05,

** p<0.01.

**Table 3.** HRs for all-cause and cardiovascular disease mortality in the study population (n=1,552)

Characteristics	All-cause mortality			Cardiovascular disease mortality		
	No. of events	Crude HR (95% CI)^1^	Fully adjusted HR (95% CI)^2^	No. of events	Crude HR (95% CI)^1^	Fully adjusted HR (95% CI)^2^
Normal weight	263 (504)	1.00 (reference)	1.00 (reference)	103 (504)	1.00 (reference)	1.00 (reference)
Slightly overweight	285 (491)	1.01 (0.86, 1.20)	1.19 (0.81, 1.76)	133 (491)	1.20 (0.93, 1.55)	1.84 (0.98, 3.45)
Overweight	199 (308)	1.17 (0.97, 1.40)	1.46 (0.95, 2.24)	84 (308)	1.25 (0.93, 1.66)	2.73 (1.40, 5.31)
Obese	172 (249)	1.35 (1.12, 1.64)	1.37 (0.86, 2.18)	86 (249)	1.70 (1.28, 2.27)	2.16 (1.03, 4.50)
Never smoker	198 (458)	1.00 (reference)	1.00 (reference)	93 (458)	1.00 (reference)	1.00 (reference)
Previous smoker	328 (558)	1.45 (1.22, 1.73)	1.63 (1.12, 2.36)	141 (558)	1.31 (1.01, 1.70)	1.77 (0.92, 3.37)
Current smoker	393 (536)	2.74 (2.31, 3.25)	3.82 (2.74, 5.33)	172 (536)	2.54 (1.97, 3.27)	5.23 (2.96, 9.24)
Hypertension	298 (441)	1.28 (1.11, 1.47)	1.32 (1.15, 1.53)	146 (441)	1.49 (1.21, 1.82)	1.51 (1.22, 1.86)
Normal eGFR						
Normal FLI	254 (474)	1.00 (reference)	1.00 (reference)	98 (474)	1.00 (reference)	1.00 (reference)
Fatty liver	351 (527)	1.43 (1.21, 1.68)	1.35 (1.09, 1.66)	158 (527)	1.66 (1.29, 2.13)	1.37 (0.99, 1.89)
Low eGFR						
Normal FLI	81 (190)	0.73 (0.57, 0.94)	0.87 (0.67, 1.12)	42 (190)	0.97 (0.68, 1.40)	1.18 (0.82, 1.70)
Fatty liver	177 (288)	1.06 (0.87, 1.28)	1.06 (0.84, 1.33)	80 (288)	1.21 (0.9, 1.63)	1.04 (0.73, 1.48)
RHF						
Normal FLI	32 (40)	2.07 (1.43, 2.99)	1.67 (1.15, 2.42)	17 (40)	2.83 (1.69, 4.74)	2.29 (1.36, 3.85)
Fatty liver	24 (33)	2.06 (1.36, 3.14)	1.96 (1.27, 3.01)	11 (33)	2.41 (1.29, 4.50)	2.23 (1.17, 4.24)

HR, hazard ratio; CI, confidence interval; eGFR, estimated glomerular filtration rate; FLI, fatty liver index score.

1 Adjusted for age only.

2 Adjusted for age, body mass index, alcohol consumption, tobacco smoking status, and hypertension.
