# Supplementary figures and images for: Renal hyperfiltration, fatty liver index, and the hazards of all-cause and cardiovascular mortality in Finnish men
Source: Epidemiol Health. 2020 Dec 24;43:e2021001. doi: 10.4178/epih.e2021001 (PMC7952838; doi:10.4178/epih.e2021001)

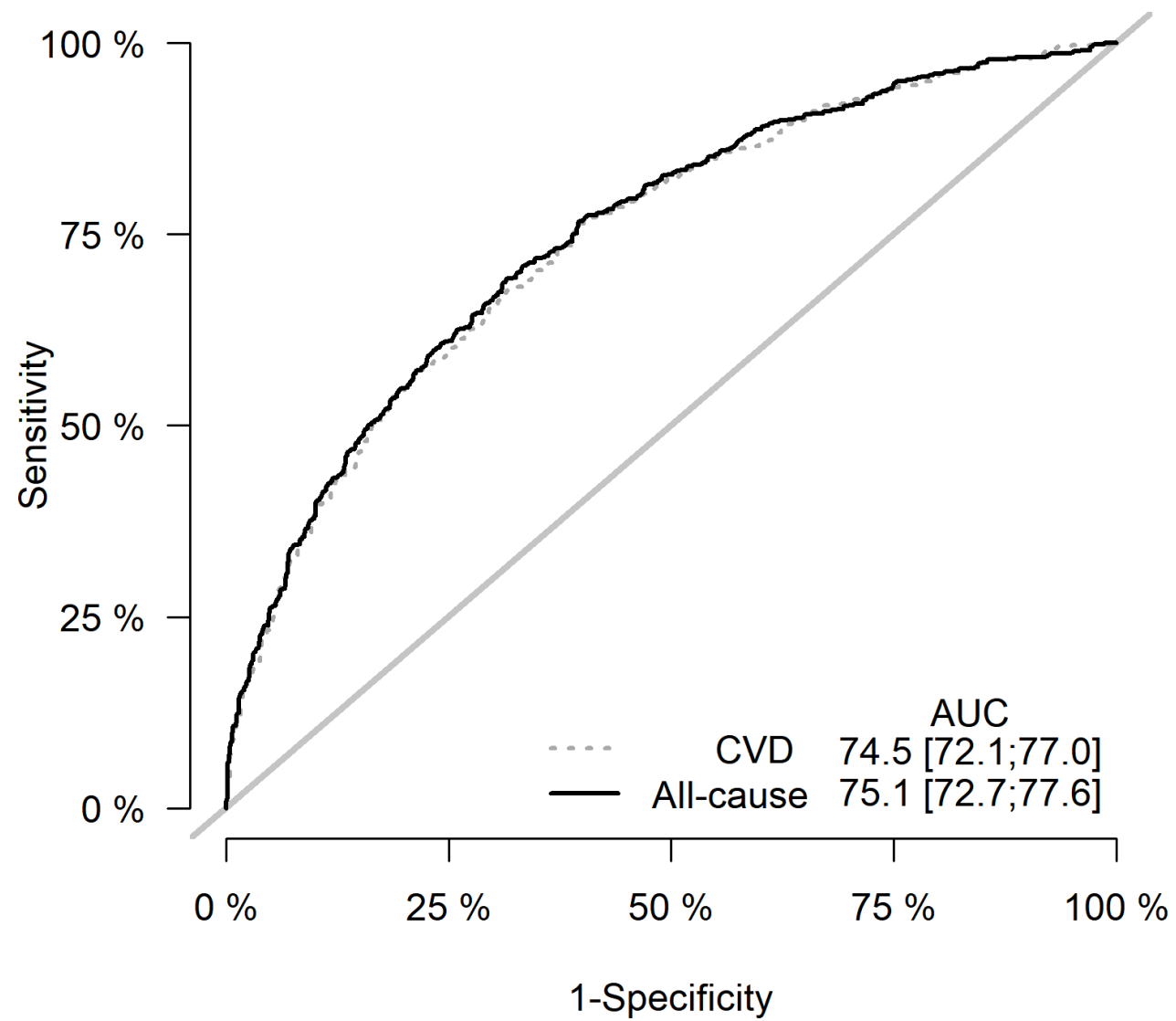

Supplement: Supplementary Material 1. [file epih-43-e2021001-suppl.pdf]
